# Climate variability supersedes grazing to determine the anatomy and physiology of a dominant grassland species

**DOI:** 10.1007/s00442-022-05106-x

**Published:** 2022-01-12

**Authors:** Seton Bachle, Jesse B. Nippert

**Affiliations:** 1 Division of Biology, Kansas State University, Manhattan, KS USA; 2 Present Address: Department of Forest and Rangeland Stewardship, Colorado State University, Fort Collins, CO USA; 3 Present Address: US Forest Service, Rocky Mountain Research Station, Rapid City, SD USA

**Keywords:** Ecophysiology, Anatomy, Climate gradient, Cattle grazing, *Andropogon gerardii*

## Abstract

Grassland ecosystems are historically shaped by climate, fire, and grazing which are essential ecological drivers. These grassland drivers influence morphology and productivity of grasses via physiological processes, resulting in unique water and carbon-use strategies among species and populations. Leaf-level physiological responses in plants are constrained by the underlying anatomy, previously shown to reflect patterns of carbon assimilation and water-use in leaf tissues. However, the magnitude to which anatomy and physiology are impacted by grassland drivers remains unstudied. To address this knowledge gap, we sampled from three locations along a latitudinal gradient in the mesic grassland region of the central Great Plains, USA during the 2018 (drier) and 2019 (wetter) growing seasons. We measured annual biomass and forage quality at the plot level, while collecting physiological and anatomical traits at the leaf-level in cattle grazed and ungrazed locations at each site. Effects of ambient drought conditions superseded local grazing treatments and reduced carbon assimilation and total productivity in *A. gerardii*. Leaf-level anatomical traits, particularly those associated with water-use, varied within and across locations and between years. Specifically, xylem area increased when water was more available (2019), while xylem resistance to cavitation was observed to increase in the drier growing season (2018). Our results highlight the importance of multi-year studies in natural systems and how trait plasticity can serve as vital tool and offer insight to understanding future grassland responses from climate change as climate played a stronger role than grazing in shaping leaf physiology and anatomy.

**Supplementary Information:**

The online version contains supplementary material available at 10.1007/s00442-022-05106-x.

## Introduction

The Great Plains is the largest expanse of grasslands in North America, reaching from Saskatchewan through Texas (Robinson et al. 2019; Jones et al. 2020). The community composition and productivity of native grasses in the Great Plains varies as a result of gradients in precipitation (longitudinally) and temperature (latitudinally) (Teeri and Stowe 1976; Sala et al. 1988; Lura et al. 2019). The impacts of these gradients are reflected in the grassland ecotones of the Great Plains (arid to mesic) that separate regions of shortgrass, mixed-grass, and tallgrass prairies (DeLuca and Zabinski 2011; Dixon et al. 2014). Each of these prairie systems are dominated by a few grass species that account for a majority of annual production. For example, *Andropogon gerardii* (tallgrass prairie) and *Bouteloua gracilis* (shortgrass steppe) can account for 70–90% of annual biomass in their respective ecosystems (Weaver 1968; Smith and Knapp 2003; Sasaki and Lauenroth 2011; Lura et al. 2019; Hoffman et al. 2020).

Dominant grasses thrive in their native habitats, because each has evolved specialized functional traits as mechanisms of persistence within each region’s disturbance regimes (Anderson 2006; Bachle et al. 2018; Jardine et al. 2021). These adaptations include but are not limited to: (1) large shallow rooting systems comprised of fine roots that quickly absorb water (Nippert and Knapp 2007; Nippert et al. 2012); (2) belowground meristematic tissues (“bud banks”) which provide new growth after senescence, fire, and grazing (Dalgleish and Hartnett 2006; Ott and Hartnett 2015; Ott et al. 2019); and (3) specialized leaf morphology and anatomy to maximize light capture and minimize water loss to decrease the drought effects (Hameed et al. 2012; Nunes et al. 2020). While these functional traits improve our understanding of the continued success of grass species in their respective region, less is understood about how these traits vary across spatial scales within a broad geographic region. For instance, which anatomical and physiological traits confer persistence locally (tallgrass prairie) and do these traits express the same relationship across different locations and climates (Great Plains)?

Trait-related investigations of dominant C_4_ grass species like *A. gerardii* have primarily focused on assessing productivity, whole-leaf economics, or genomics/phenotypes (Avolio and Smith 2013; McAllister et al. 2015). Much of this research was built around a common garden experimental design, and has yielded many novel insights such as the genotypic changes in local populations (ecotypes) across regions (Mendola et al. 2015; Maricle et al. 2017; Galliart et al. 2019). In addition, other studies have determined that large intraspecific variation in functional traits regulating physiology exists in *A. gerardii*, enabling a single species to occupy a wide geographic and environmental breadth (Bachle and Nippert 2018, 2021; Bachle et al. 2018; Westerband et al. 2021). To date, investigations of genotypic and physiological variability in *A. gerardii* have provided key perspectives on population-level plasticity across naturally occurring precipitation gradients (Avolio and Smith 2013; McAllister et al. 2015). Understanding the relationship between anatomical and physiological traits within populations across multiple years may provide a more detailed understanding of how a single species responds to future climate variability. In addition, the intraspecific variation in a species’ physiology across these climate gradients may be heavily influenced by anatomical traits, which are currently poorly understood.

Anatomical traits are often not assessed because of the tedious and labor-intensive preparation and data collection, but information gleaned from these methods allows for deeper understanding of physiological mechanisms (Wahl and Ryser 2000; McElrone et al. 2004; Carmo-Silva et al. 2009). Plant physiology has typically been constrained by variation in anatomical traits, because the structural framework of tissue architecture sets limits for physiological function (Esau 1939; Furbank 2016; Bellasio and Lundgren 2016). For instance, the innovation and diversification of xylem affect survival in drought conditions across functional types (Scoffoni et al. 2014; Hammond et al. 2019; Ocheltree et al. 2020). Also, alterations to stomatal anatomy and densities regulate water-usage, because stomata serve as the gateway for the flux of CO_2_, O_2_, and H_2_O to and from the leaf. This regulation is essential, because CO_2_ and H_2_O fluxes directly impact both carbon and water balance at the leaf-level, and the subsequent whole organism performance. In addition to carbon and water, nitrogen is also necessary for proper physiological functioning, all of which are required for cellular upkeep and development of anatomical tissues (Chaves et al. 2003; Lundgren and Fleming 2019).

Investigations focused on the anatomical changes of relatively few species associated with different levels of carbon, nitrogen, and water availability are typically done in greenhouses or in agricultural settings and only focus on few traits—usually for the purpose of yield enhancement (Henry et al. 2012; Retta et al. 2016; Ermakova et al. 2019). While the importance of this research should not be overlooked due to its significance in feeding a growing global population, these data are collected from controlled environments with abundant resources and such results may not apply to natural ecosystems. Under field conditions, resources for native species are typically variable and often limiting. In addition, morphological responses from annual agricultural species do not always translate to regions like the Great Plains, which is comprised of native perennial grasses that invest beyond a single annual reproductive cycle (Benson et al. 2004; Benson and Hartnett 2006). Currently, 60% of the Great Plains is now at risk of, or has previously been degraded due to anthropogenic pressures (e.g., agriculture and climate change) (Olimb and Robinson 2019), and the perennial grasslands that remain are used for cattle grazing. To our knowledge, a multi-year investigation across a climate gradient to assess the effects of climate in conjunction with cattle grazing on leaf-level anatomy and physiology has not been done for a native grass. This type of study will aid in determining mechanistic strategies at the leaf-level that constrain physiological responses to ecological drivers in the Great Plains.

Here, we investigate naturally occurring populations in their home environments under a range of environmental conditions. This approach allows for an assessment of responses to climate variability within a site and comparisons of variability across sites. This study aims to provide a mechanistic understanding of how varying climate and grazing impacts a dominant species’ (*A. gerardii*) physiological and anatomical traits across a latitudinal gradient in the Great Plains. We hypothesized that: (1) due to site-level differences in climate histories across the latitudinal gradient, and contrasting growing season conditions in 2018 and 2019, there would be significant differences in mean and variability (measured here as the coefficient of variation) of leaf-level nutrient content, anatomical traits, and instantaneous physiological responses across sites; (2) because anatomical traits constrain physiological responses to water availability, the existing trait relationships will show significant differences between years sampled due to the disparity in precipitation received; and (3) due to the stress of compensatory growth and reallocation of resources, cattle grazing will emphasize leaf-level anatomy and nutrient content differences between treatments and across locations.

## Materials and methods

### Site descriptions

This experiment was conducted at three separate locations dominated by *A. gerardii* within the tallgrass prairie region of the Great Plains during the 2018 and 2019 growing seasons. These locations include: (1) a Long-Term Ecological Research site (LTER), Konza Prairie Biological Station (Northern Kansas site: N. KS) located in the northern Flint Hills region of eastern Kansas USA (39.1 °N, 96.9 °W), (2) the Flint Hills Prairie Preserve (Southern Kansas site: S. KS) located at the mid-point of the Flint Hills region (38.2 °N, 96.3 °W), and (3) the Platte River Prairies (Nebraska site: NE) located within the Big Bend region of south-central Nebraska USA (40.4 °N, 98.3 °W). All sites are owned by The Nature Conservancy (TNC) of Kansas and Nebraska. Data were collected from five 1-m^2^ plots equally distributed in cattle grazed and ungrazed locations across similar topographic positions (*N* = 10 plots at each site). The S. KS site was burned in the summer of 2017, but not in 2018 or 2019, and grazed at 3 animal units (AU) acre^−1^ (7.32 AU ha^−1^). This site is predominantly silty-clay soils that receives ~ 950 mm year^−1^ precipitation. Two separate experimental watersheds were utilized at N. KS, including the ungrazed watershed ‘2D’ and the grazed watershed ‘3CB’ (8 AU acre^−1^ or 19.5 AU ha^−1^), both of which were burned in 2019. N. KS receives ~ 870 mm of annual precipitation and is characterized by silty-clay soils (Bachle and Nippert 2021). Experimental plots at the NE site were located in ungrazed and grazed pastures (8 AU acre^−1^ or 19.5 AU ha^−1^); both locations were burned in the spring of 2019. The NE site receives ~ 670 mm year^−1^ with predominantly sandy soils. In 2018, the S. KS and N. KS experienced a drought that drastically reduced rainfall in the early (April–May) and mid-growing season (June–July).

### Leaf physiology and anatomy

Gas-exchange rates were measured using a Li-Cor model LI-6400XT (Li-COR Biosciences, Lincoln, NE, USA) equipped with an LED light source (maintained at 2000 µmol m^−2^ s^−1^). CO_2_ concentration was set at 400 ppm and relative humidity in the chamber was maintained between 40 and 60%. Measurements were collected between 10:00 and 14:00 CDT to collect photosynthetic rates (*A*_n_), stomatal conductance (*g*_s_), and transpiration rates (*E*) during two periods (June and August) in the 2018 and 2019 growing seasons. At each sampling period, leaves from three individual *A. gerardii* grasses were measured in each plot. To avoid confounding results due to leaf age, only new and fully expanded leaves were used for analyses. Measurements were recorded when gas-exchange levels remained stable for ~ 2 min. These same individual leaves were also used to determine nutrient content and anatomical traits within each growing season.

**Fig. 1:**
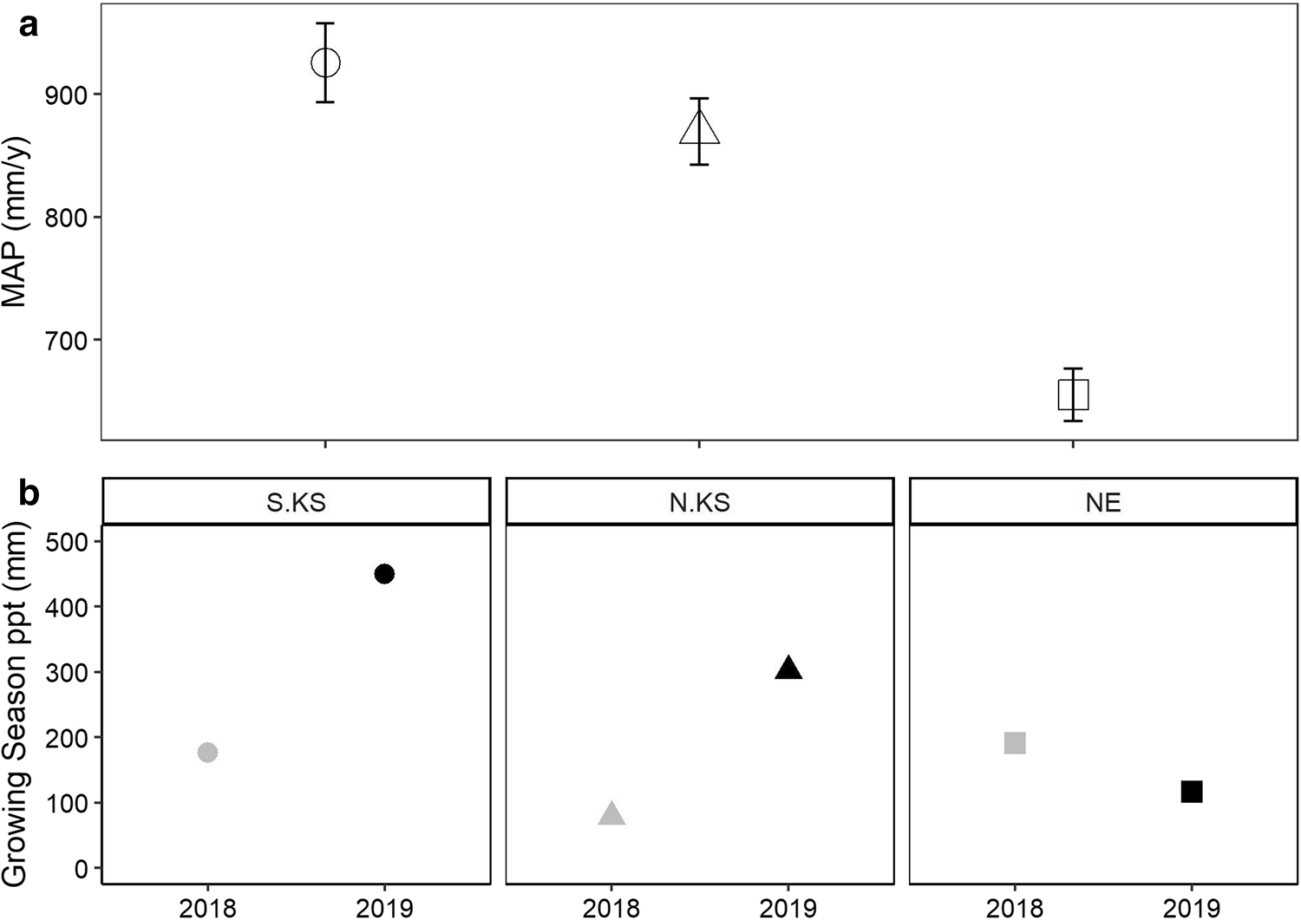
**a** Long-term mean annual precipitation for each location (1981–2019); error bars represent standard error. **b** Growing season precipitation from May 1st to August 10th during 2018 and 2019. Shapes denote location (open circle, S. KS; open triangle, N. KS; open square, NE), while color denotes year (gray, 2018; black, 2019)

Following physiological gas-exchange measurements, the previously measured leaf tissues were then clipped (~ 30 mm) and immediately placed into FAA (10% formalin/5% glacial acetic acid/50% ethanol) (95% EtOH/35% DI water) for vacuum infiltration to analyze anatomical traits. Leaf tissues were then cross sectioned to a 4 µm thickness with a Leica RM2135 microtome (Leica Biosystems, Newcastle, UK), stained with Safranin-O and Fast Green (Ruzin 2000), and imaged at 100X and 200X on an Olympus BH-2 compound microscope (Olympus America Inc, Melville, NY) (Fig. 1). We then quantified anatomical traits using IMAGEJ software (Rasband 1997) and the procedure detailed by Bachle and Nippert (2018). The selected anatomical traits included: the total cross-sectional area measured (TMA), bundle sheath cell area (BS_A,_), mesophyll area (MS_A_), bundle sheath: mesophyll area (*BS:MS*), bulliform area (*B*_A_), xylem area (X_A_), and xylem reinforcement (*t/b*), which is the ratio of xylem wall thickness (*t*) with xylem diameter (*b*). The following traits were measured on an area basis (as a percentage of TMA): BS_A_, MS_A_, *B*_A_, and *V*_A_. In addition, due to the small size of minor veins in the sampled leaf tissue, xylem characteristics were restricted to the major vascular bundles.

### Leaf stoichiometry and biomass

Carbon (C) and nitrogen (N) contents were measured on the same leaves used for gas-exchange. These leaves were dried and ground for elemental composition of carbon and nitrogen per plot (protocol outlined in Connell et al. 2020). Aboveground biomass was determined by clipping herbaceous tissues in one 0.1 × 0.1 m frame per plot at the conclusion of each growing season. This biomass was sorted to exclude dead biomass (when necessary) and then dried at 60 °C for 48 h and weighed to determine dry mass.

### Statistical analyses

All analyses were completed in the statistical program R V3.5.3 (R Core Team 2020). We evaluated homogeneity of variances by examining residuals vs fitted, normality using *qq*-plots and, when necessary, a Shapiro–Wilk’s test. TMA and *t* were the only traits that required non-parametric analyses (after attempting log transformations) via Kruskal–Wallace test accompanied with a post hoc pairwise Wilcox test. To assess the effects of grazing and climate differences between locations, we utilized repeated-measures mixed-effects model ANOVAs with separate models for each physiological, anatomical, and nutrient trait as the response variables, and location, grazing treatment, and year sampled as predictor variables, and plot as the random effects. We reported the *F* values and binned the *P* values by levels of significance (Table 1). Tests were performed using the “lmer” function within the “lmerTest” package (Kuznetsova et al. 2017). We also performed a principal component analysis (PCA) using the “prcomp” function within the “stats” package on the mean trait data across locations, grazing treatments, and years to summarize the relationships and range of physiological, functional, and anatomical responses. We did not include traits such as *t* (xylem thickness) and *b* (xylem diameter) as they are already components of xylem reinforcement (*t/b*). In addition, we did not include climate parameters here; rather, we focused on key traits coming from our predictor variables. Standard deviations, proportion of variance, cumulative proportions, and loading scores of principle components are located in Table S3.

**Table 1:** ANOVA results, reported as *F* values for leaf-level physiological, anatomical, stoichiometric traits, and biomass

Trait	Location	Grazing treatment	Year	*L* × *T*	*L* × *Y*	*T* × *Y*	*L* × *T* × *Y*
*A*_n_	26.91***	0.79	280.30***	2.74ˆ	23.79***	5.14*	0.23
*g*_s (SQRT)_	11.25***	0.37	356.52***	3.19	49.03***	0.00	1.52
*E*	5.73**	0.08	1.05	1.48	51.92***	7.61**	1.92
TMA _(LOG)_	5.25**	1.27	0.33	0.57	0.80	0.99	0.91
BS_A_	10.45***	1.21	191.00***	2.84ˆ	15.19***	4.67*	0.39
MS_A_	1.05	7.46*	1.70	7.30***	4.21*	3.09ˆ	0.40
*V*_A_	1.52	4.99ˆ	9.46**	4.59*	19.73***	8.15**	4.53*
BS:MS	6.07**	0.33	61.63***	2.00	8.24***	6.25*	0.37
*B*_A_	0.04	0.32	62.08***	1.94	19.88***	6.00*	1.70
*X*_A_	1.03	0.17	14.00***	4.96**	3.00ˆ	9.97**	8.19***
*t*/*b*_(LOG)_	1.68	2.10	31.08***	7.05**	4.94**	1.03	6.08**
*N*	6.66***	2.91	137.86***	1.05	1.14	0.04	0.58
C:N	11.36***	4.75*	73.38***	2.35ˆ	0.58ˆ	0.06	3.57*
Biomass _(LOG)_	57.15***	67.19***	5.52*	3.09ˆ	2.49ˆ	7.50**	0.58

Subscript text in parentheses refers to data transformation necessary to meet assumptions of normality. ˆ*P* < 0.10, *P < 0.05, **P < 0.01, ***P < 0.001

*A*_n_, photosynthetic rate; *g*_s (SQRT)_, transformed stomatal conductance; *E*, transpiration rate; TMA_LOG_, log-transformed total measured area; BS_A_, bundle sheath area; MS_A_, mesophyll area; *V*_A_, vein area; BS:MS, ratio of bundle sheath to mesophyll area; *B*_A_, bulliform cell area; *X*_A_, xylem area; *t*/*b*_(LOG)_, log-transformed xylem reinforcement; N, nitrogen content; C:N, ratio of carbon-to-nitrogen content; biomass_(LOG)_, log-transformed biomass

## Results

### Leaf-level physiological traits

**Fig. 2:**
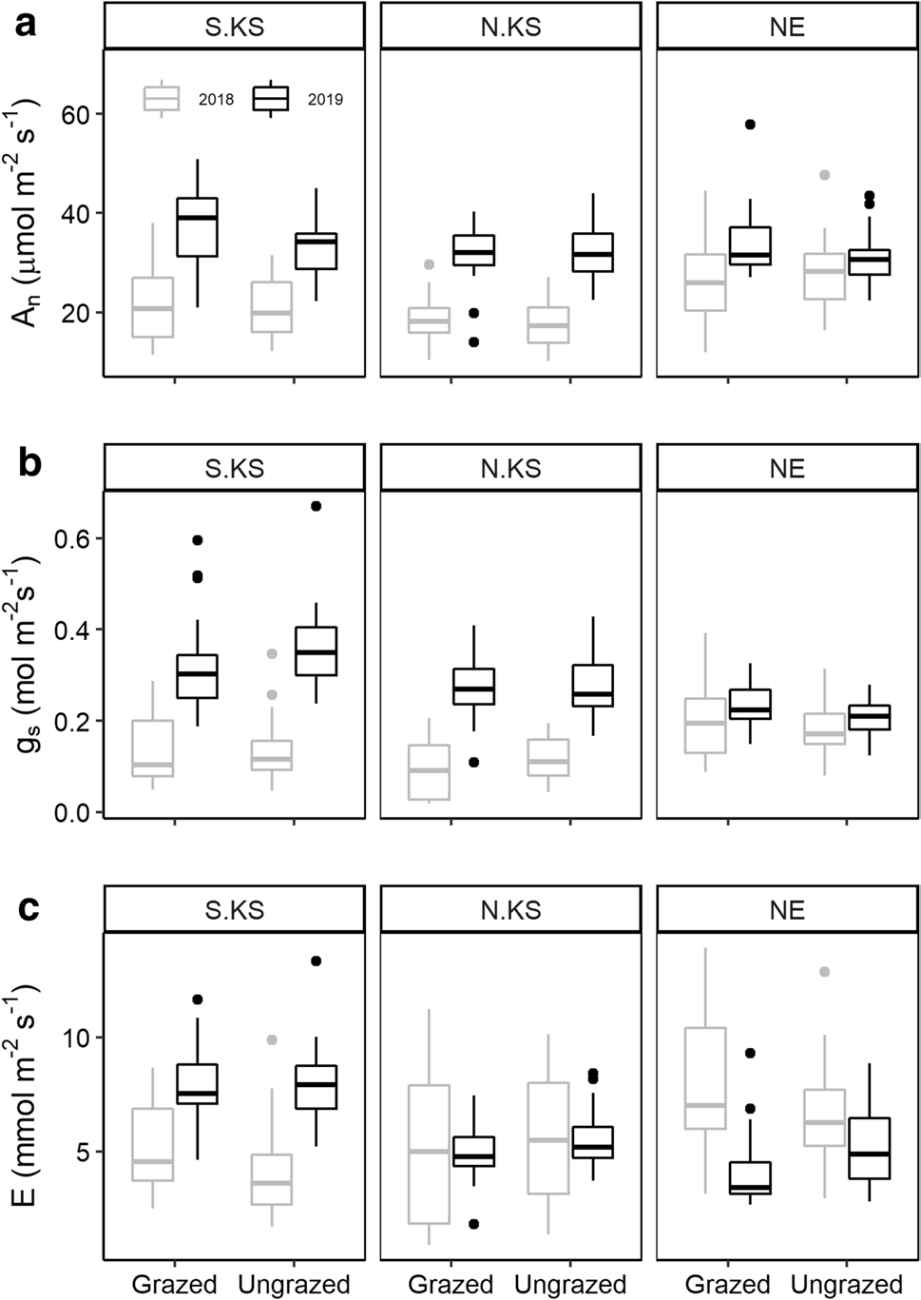
Gas-exchange collected at each site and treatment during the 2018 (gray) and 2019 (black) growing seasons. **a** Photosynthetic rate (*A*_n_); **b** stomatal conductance to vapor (*g*_s_); **c** transpiration rate (*E*). Thickened lines represent the median value; upper and lower edges of box represent the interquartile values (25th and 75th percentiles)

Leaf-level gas-exchange in *A. gerardii* varied by location (*P* < 0.005), but not by grazing treatment (*P* > 0.40) (Fig. 2; Table 1). *E* was statistically similar across the years sampled (*P* > 0.05; Fig. 2C; Table 1). Grasses at NE had the highest gas-exchange rates in 2018, while S. KS displayed the highest rates in 2019 (Fig. 2). *A*_n_ and *g*_s_ increased between 2018 and 2019 (*P* < 0.001), most notably at S. KS (74% and 156%, respectively) and N. KS (119% and 150%, respectively) (Fig. 2A, B). In addition, there was an interaction between location and year sampled for both *A*_n_ and *g*_s_ (*P* < 0.001; Table 1). While there was no statistically significant latitudinal trend for *E* and *g*_s_ discernible in 2018 (Fig. 2B, C), *A*_n_ was observed to increase with growing season precipitation and a decrease with growing season temperatures regardless of grazing.

### Internal anatomical leaf traits

The total measurable area of internal anatomical traits (TMA) varied significantly among locations (*P* < 0.05) but remained statistically similar across grazing treatment and year (*P* = 0.29; 0.57, respectively; Table 1). Specifically, TMA at S. KS was significantly smaller compared to other locations in 2019 (*P* < 0.05; Table 1**).** Additionally, BS_A_ was displayed an interaction between the location and year sampled, with a significant increase in BS_A_ from 2018 to 2019 (*P* < 0.001; Table 1). In 2018, BS_A_ in samples from N. KS was significantly higher than either S. KS or NE; however, S. KS samples contained the highest BS_A_ in 2019 (*P* < 0.05; Table 1). In addition, C:N ratios, MS_A_, and biomass were the only traits that were affected by the grazing treatment, but only within N. KS in 2019 (*P* < 0.05; Table 1). Overall, MS_A_ did not change between years nor among locations (*P* > 0.05), maintaining ~ 40% of TMA.

**Fig. 3:**
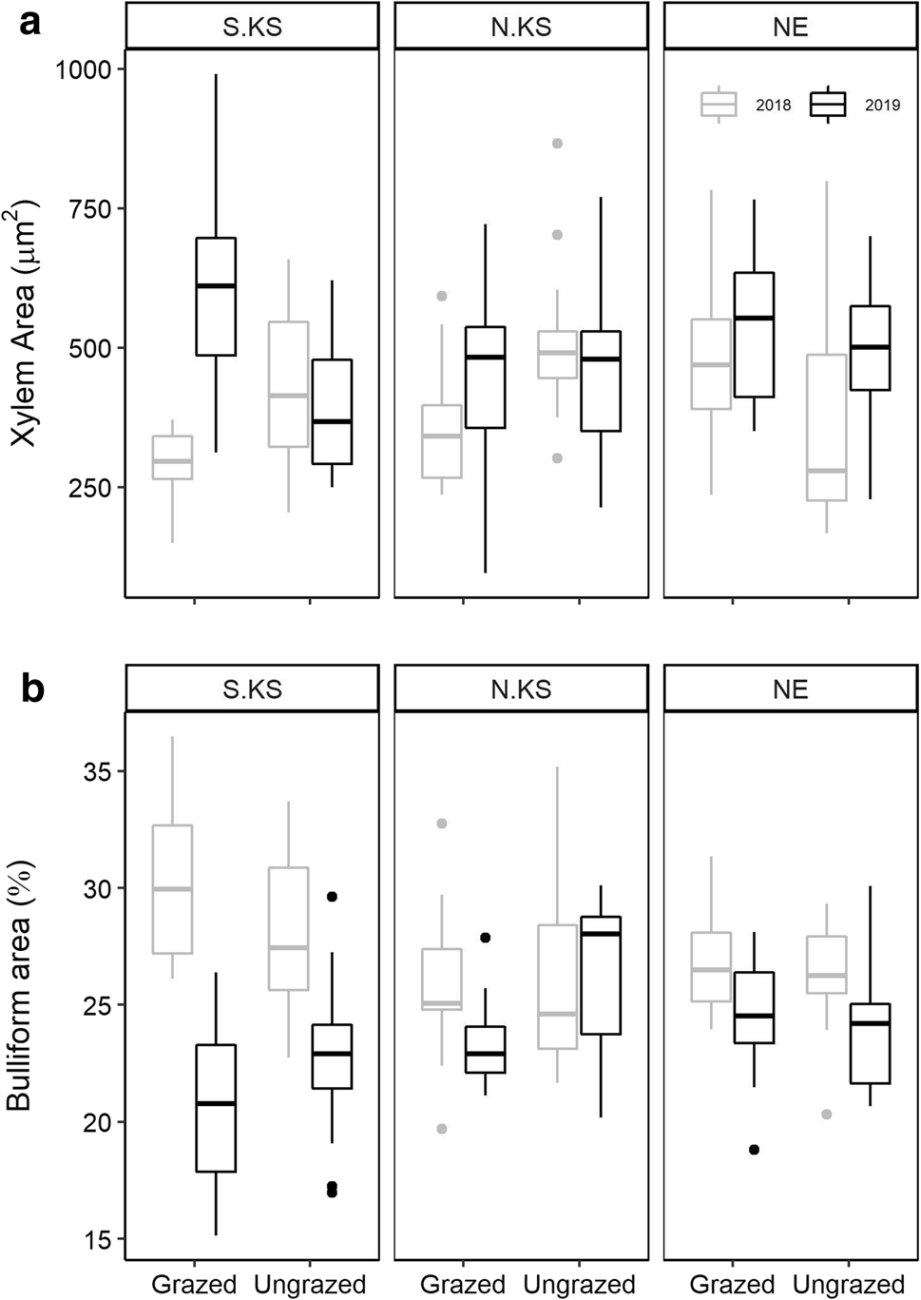
Boxplots of anatomical data collected at each site and treatment during the 2018 (gray) and 2019 (black) growing season. **a** Mean xylem area (*X*_A_); **b** mean bulliform cell area (*B*_A_). Thickened lines represent the median value; upper and lower edges of box represent the interquartile values (25th and 75th percentiles)

The ratio of bundle sheath area and mesophyll area (BS:MS) displayed significant effects from location, year, and their interaction (*P* < 0.03, *P* < 0.0001, *P* < 0.0001; Table 1). *V*_A_ varied significantly between years (*P* < 0.05), but was not affected by grazing or location sampled (*P* > 0.05, *P* = 0.056; Table 1). *V*_A_ at S. KS and NE increased from 2018 to 2019; in contrast, *V*_A_ at N. KS decreased (Table S2). Tissues within *V*_A_ were consistently between 12 and 18% of TMA (Table S2). *B*_A_ did not vary across locations (*P* = 0.96) or grazing treatment (*P* = 0.59; excluding N. KS in 2019), but significantly decreased from 2018 to 2019 in all locations except N. KS (*P* = 0.25; Fig. 3B; Table 1). In addition, TMA consisted of ~ 20–30% *B*_A_ across each location, year, and grazing treatment (Table S2). *X*_A_ also increased across years sampled (Fig. 3; *P* < 0.005) but remained statistically similar across locations and grazing treatment (*P* = 0.36, *P* = 0.69, respectively; Table 1). S. KS was the only location that exhibited a large difference in *X*_A_ between control and grazing treatment in both 2018 and 2019 (*P* < 0.02, *P* < 0.005, respectively), while grazing only impacted *X*_A_ at N. KS in 2018 (*P* < 0.05; Fig. 3A; Table 1). Finally, xylem reinforcement (*t/b*) followed a similar pattern to *X*_A_ and significantly decreased across years sampled (Table 1; *P* < 0.005), but did not differ across locations or grazing treatments (*P* > 0.05; Table 1). Microanatomical traits displayed similar general trends with growing season temperature and climate as leaf-level physiological traits; however, our results also reflect an increased coordination between internal leaf tissues specific to water-use.

### Stoichiometry and productivity

**Fig. 4:**
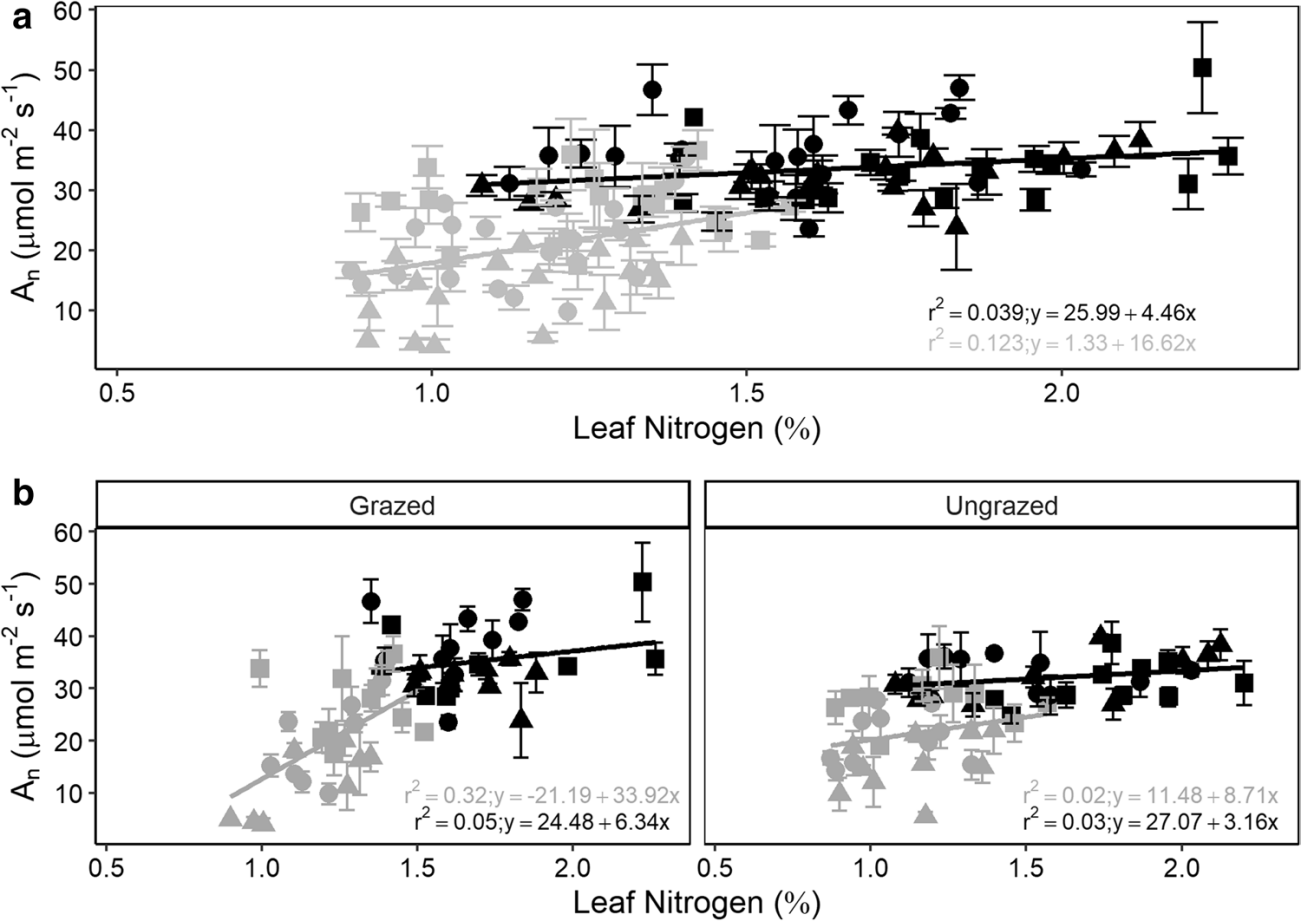
Linear regression relating leaf-level nitrogen content and mean photosynthetic rate at each location and year (mean ± SE). **a** Relationship across years; **b** relationship separated by treatment. Shapes denote location (circle, S. KS; triangle, N. KS square, NE), while color denotes year (gray, 2018; black, 2019)

Carbon and nitrogen contents in *A. gerardii* leaves varied according to year and location, but C:N was the only stoichiometric measurement affected by the grazing treatment (*P* > 0.05; Table 1). Leaf nitrogen content was consistently higher in 2019 than in 2018 (*P* < 0.0001; Table 1); grass leaves at NE had the highest nitrogen content, regardless of year (Table S1). In addition, C:N ratios were higher in 2018 than 2019 and varied by location sampled and grazing treatment (*P* < 0.05; Table 1). The C:N ratio was higher at both S. KS and N. KS relative to NE in both years sampled, regardless of treatment (Table S1). Aboveground biomass varied by location, year, and grazing treatment (*P* < 0.05; Table 1). NE was the most productive location in both 2018 and 2019, in both grazed and ungrazed plots (Table S1). N was observed to increase positively with traits specific to carbon assimilation including BS_A_ and *A*_n_ in both grazed and ungrazed treatments (Fig. 4).

### Trait relationships and variation

**Fig. 5:**
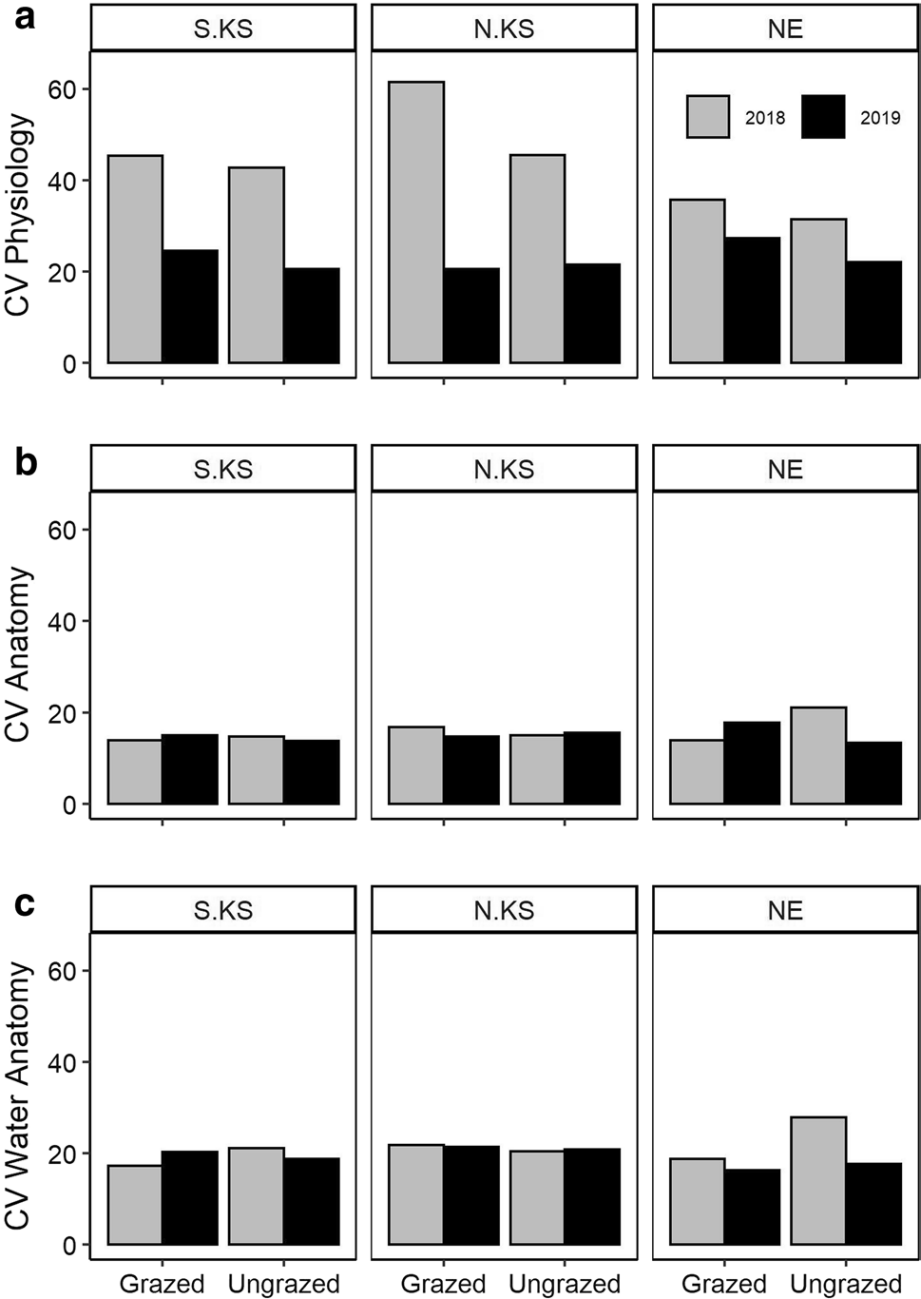
Coefficient of variation (CV) at each location and year. **a** Combined mean CV for the photosynthetic rate (*A*_n_)*,* stomatal conductance (*g*_s_)*,* and transpiration rate (*E*). **b** Combined mean CV for all anatomical traits (excluding redundancies). **c** Combined mean CV for anatomical traits that influence water storage or transport (*X*_A_*, t/b, B*_A_). Colors denote year of sampling (gray, 2018; black, 2019)

**Fig. 6:**
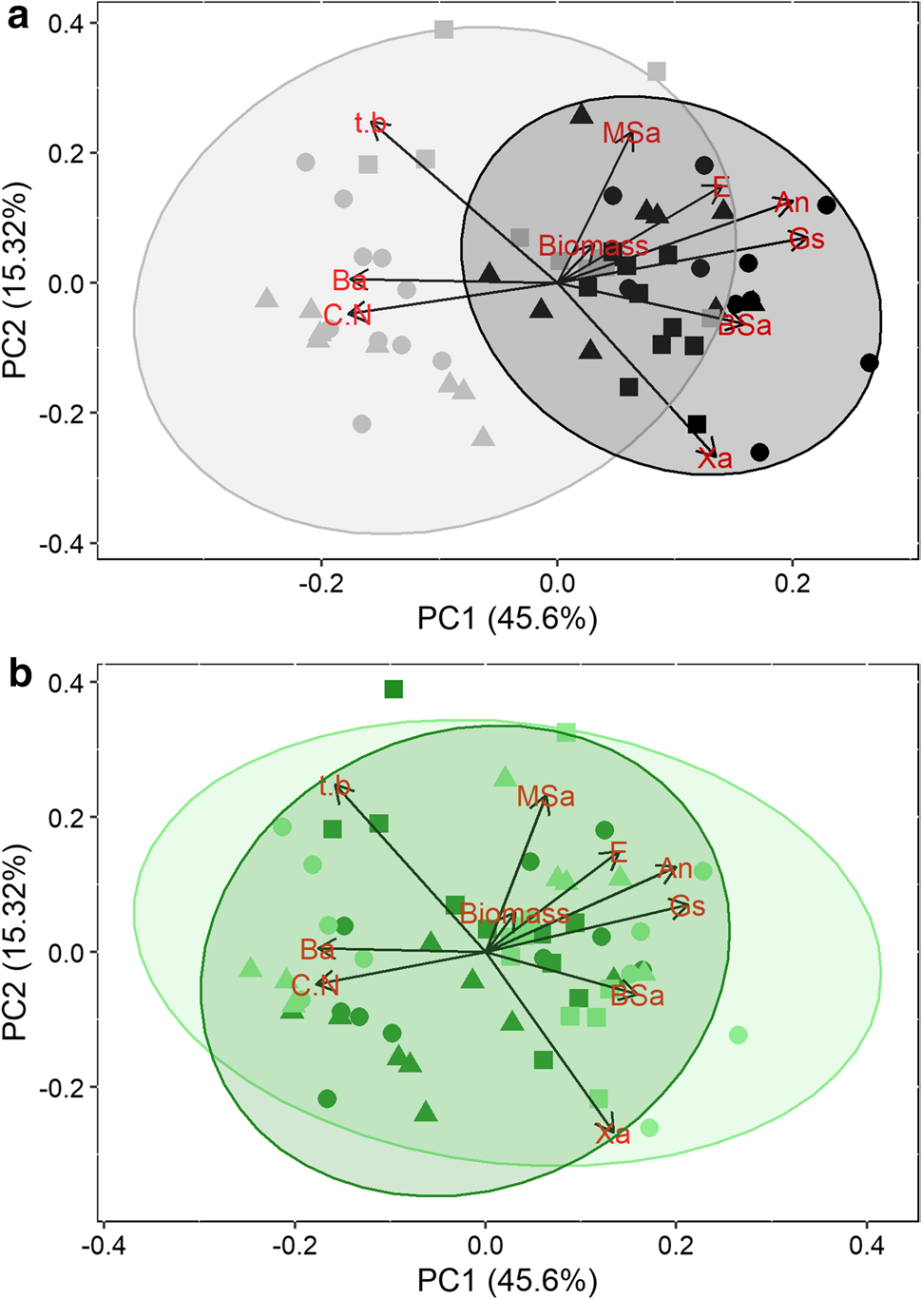
Principal components analysis (PCA) of mean trait values for *A. gerardii* at each location and year. This plot provides a summary of populations in multivariate trait space using the first two PC axes, which together account for 60.9% of the trait variation. **a** Grouped across years; **b** grouped by treatment. Shapes denote location (circle, S. KS; triangle, N. KS square, NE), while color denotes grouping: **a** gray, 2018; black, 2019. **b** Light green, grazed; dark green, ungrazed. Information concerning PCA axes importance and subsequent loadings are located in Table S3

While traits did show relationships with average climate parameters (MAP and MAT) for the three sites, grazing had little effect on most traits and relationships (Table S1, S2). However, higher temperatures were associated with lower N content, higher C:N ratios, and decreased gas-exchange rates (Table S1). Trait data collected across locations, years, and grazing treatment displayed statistically significant variation (Table S1, S2). The mean coefficient of variation (CV) in physiological traits (*A*_n_, *g*_s_, and *E*) was significantly higher than the mean CV in anatomical traits (Fig. 5A, B). However, water-usage/storage traits (*X*_A_*, t/b*, and *B*_A_), were responsible for the majority of anatomical variation (Fig. 5C). In addition, slight changes in anatomical CV were observed between years and locations, while physiology displayed significantly higher CV in 2018 than 2019 (Fig. 5). According to our PCA analyses, axis 1 and 2 cumulatively explained 60.9% of the variation in traits (Fig. 6). Physiological and anatomical trait relationships were more distinct when grouped by year (Fig. 6A) than grazing treatment (Fig. 6B), which revealed higher gas-exchange rates, BS_A,_ MS_A_, and X_A_ with increased rainfall received in 2019, while traits associated with the drier 2018 included *t/b,* C:N, and *B*_A_ (Fig. 6A). In addition, there was increased dispersion in 2018 and sites were also more clustered in 2018 when compared to 2019 (Fig. 6B).

## Discussion

Climate histories in grassland ecosystems are often variable compared to other biomes (Zhang et al. 2010; Knapp et al. 2015; Flanagan et al. 2017). Our data illustrated that the pattern of variation in response to wet/dry years was not uniform across locations and these responses to interannual climate had a larger effect than responses to cattle grazing. Here, our results emphasize the large differences in physiological and anatomical responses that can exist within a widespread C_4_ grass species (*A. gerardii*) across multiple years and locations with distinct climate (precipitation and temperature) and variable management histories (i.e., grazing) (Fig. 1).

A large number of studies have investigated how the dominant C_4_ grass species *A. gerardii* responds to changes in precipitation (Knapp 1985; Dietrich and Smith 2016; Hoffman et al. 2018). However, a few studies have compared responses to multiple key grassland ecosystem drivers (fire, climate, and grazing), which have been repeatedly shown to impact physiological responses and biomass (O’Keefe and Nippert 2017; O’Connor et al. 2020; Connell et al. 2020). In agreement with our first hypothesis, significant differences in leaf-level physiology, anatomy, stoichiometry, and biomass were observed across sites and between years in this study. The long-term climate histories of each location (Fig. 1) were responsible for shaping functional traits of local populations, allowing for site-specific leaf-level anatomy and physiology (Figs. 2, 3; Tables 1, S1, S2) (Hoffman and Smith 2020; Bachle and Nippert 2021). The drought conditions during 2018 at both Kansas locations resulted in significantly reduced photosynthetic rates, stomatal conductance, and leaf nitrogen content (Fig. 2; Tables 1, S1). Increasing aridity and water stress decreases stomatal aperture, allowing for reduced water loss at the leaf-level; however, long durations of water stress can lead to carbon starvation (Lawson and Matthews 2020; Nunes et al. 2020). Similarly, decreased *X*_A_ and increased *B*_A_ were also observed in 2018 (Fig. 3), reflecting changes in water-use strategies. Previous research indicates that increased *X*_A_ allows for greater water transport, but it also increases the likelihood of cavitation during droughts or when the water column is under high tension (Olson et al. 2020). Therefore, *A. gerardii* may be coordinating both instantaneous (gas-exchange) and structural/investment (anatomical) mechanistic strategies in response to decreased water availability.

Intraspecific trait variability (CV) was statistically different between years, supporting our first hypothesis, but relatively similar across locations (Fig. 5). This result is surprising due to the different climatic and management histories of each location (Fig. 1). The greatest variation was reported for gas-exchange measurements (*A*_n_, *g*_s_, *E*) in 2018, which were ~ 2 times higher than the following year (at both S. KS and N. KS) (Fig. 5A). While high variability may be inherent to the instantaneous nature of gas-exchange measurements, the CV of physiological responses in 2019 was similar to all anatomical traits regardless of function (Fig. 5B, C). This decrease in physiological CV may indicate a baseline physiology, and associated physiological plasticity of *A. gerardii*, when water is less limiting. Mean anatomical traits varied significantly between 2018 and 2019, and there was little change in variability (CV) across years or grazing treatment (Fig. 5B, C). In fact, most anatomical variation resulted from water-specific traits (*X*_A_, *t/b*, *B*_A_) (Fig. 5C). This diversity in functional trait responses has been previously reported to protect individuals and populations from detrimental effects of drought (Mori et al. 2013; Kreyling et al. 2017; Roberts et al. 2019). In addition, such variation may also aid in protecting populations from the potentially negative effects from grazing.

While previous research has indicated that anatomical traits can influence/constrain physiological responses to changes in water availability (Christin et al. 2013; Guha et al. 2018; Edson-Chaves and Graciano-Ribeiro 2018; Wargowsky et al. 2021), a few studies have analyzed physiology, stoichiometry, and anatomy of the same leaf across multiple years and locations. The importance of this sampling technique allowed us to analyze relationships of both functional trait mean and variability (*CV*) (Figs. 4, S1; Tables S1, S2). These results emphasize how disparate climates across years (i.e., 2018 and 2019) can result in dissimilar relationships among and between traits and climate variables (Fig. 4; Tables S1, S2), thereby supporting our second hypothesis. For instance, *A. gerardii* photosynthetic rates correlated positively with increasing leaf nitrogen content (Fig. 4A) when analyzed between years. However, this seemingly tight relationship breaks down when analyzing each year and treatment separately (Fig. 4B), emphasizing the importance of multi-year studies. PCA results also indicate the importance of a multi-year experimental design to reveal mechanistic trait responses and relationships to contrasting growing seasons (Fig. 6). While it is well understood that higher rainfall can correspond to increased carbon assimilation observed in gas-exchange rates and biomass (Fig. 6A), the more interesting results can be seen at the anatomical level. For instance, our data indicate that tissues responsible for water conservation and water allocation/usage were diametrically opposed across 2018 and 2019. Larger xylem area (*X*_A_) is beneficial in years with more rainfall, while *A. gerardii* would benefit with increased water storage (*B*_A_) and the strengthening of the xylem walls (*X*_A_) during dry years (Fig. 6A). Increasing *t/b* provides a more rigid conduit that decreases the likelihood of cavitation under drought stress and may also explain the increased C:N as more carbon investment would be necessary to thicken xylem walls (Mauseth 1988). In addition to total growing season precipitation, the timing of precipitation is known to impact grassland productivity (Nippert et al. 2006; Craine et al. 2012), which is ultimately a result of altered anatomy and physiology (Fay et al. 2002; Wang et al. 2016; Lemoine et al. 2018). For example, high early growing season precipitation results in larger vessel areas with greater transport potential following spring rains. In contrast, early season droughts can constrain tissue develop and result in smaller vessel areas (example in Fig S1), which may reduce productivity across the growing season (Mauseth 1988).

Similar to climate variability and fire, responses to grazing are typically examined at the community or ecosystem levels, while less is understood about the physiological and anatomical mechanisms responsible for those responses (O’Keefe and Nippert 2017). However, grazing and other forms of herbivory have been previously observed to increase gas-exchange rates to compensate for the loss of tissue (Pinkard et al. 2011; O’Connor et al. 2020), which can allow for increased carbon assimilation but also increase water loss (Bertolino et al. 2019). Contrary to our expectations (hypothesis 3), we observed few statistically significant responses attributed to grazing. Gas-exchange rates within cattle grazed locations in this study were nearly identical to ungrazed locations (Table 1; Fig. 2), even during the drier 2018 growing season. In addition, only three functional traits were impacted by the grazing treatment: MS_A_, C:N ratios, and biomass production (Table 1); all of which correspond to an increased leaf size. Cattle grazing did impact functional trait variability, but only during the 2018 growing season and only in physiological and water-use anatomical trait CV (Fig. 5). The overall lack of grazing response may be due to several factors including: (1) stocking rates at each location may not be intensive enough to reflect substantive grazing pressure; (2) the experimental design may not have adequately covered/represented each site and subsequent treatment; (3) due to the evolutionary history of *A. gerardii* in the Great Plains, a heightened grazing intensity may be necessary to induce alternative physiological responses. One such adaptation may include the innately large rooting systems of native grass species which that absorb water efficiently and can serve as carbon reserves during drought (Weaver and Darland 1949; Blair et al. 2014), which could have compensated for the stress exhibited aboveground.

Our results highlight how trait plasticity can serve as an important tool for understanding the anatomical and physiological mechanisms that facilitate wide distributions of a dominant grass species exposed to varying management strategies (i.e., cattle grazing). Drought conditions in 2018 resulted in decreased gas-exchange rates and subsequent biomass production, irrespective of grazing. However, increased water availability in 2019 facilitated higher gas-exchange rates and the doubling of aboveground biomass. In addition, there was significant variation in anatomical traits across locations and between sampling years. Such leaf construction strategies frame instantaneous physiological responses to climate variability, and also other grassland drivers (i.e., grazing and fire). Results from this study underlie the importance of collecting multiple years of data from native species in natural environments. Our data also emphasize the need for increased anatomical research, as we clearly demonstrate site and climate-specific leaf construction strategies are important for understanding and contextualizing physiological responses in a dominant grass species.

## Supplementary Information

Below is the link to the electronic supplementary material.

Supplementary file1 (DOCX 911 kb)

## Data Availability

The datasets used and/or analyzed during the current study are available from the corresponding author on reasonable request.
