# Supplementary material for: Climate variability supersedes grazing to determine the anatomy and physiology of a dominant grassland species
Source: Oecologia. 2022 Jan 12;198(2):345–55. doi: 10.1007/s00442-022-05106-x (PMC8858925; doi:10.1007/s00442-022-05106-x)
Supplement: Supplementary file 1 — Supplementary file1 (DOCX 911 kb) [file 442_2022_5106_MOESM1_ESM.docx]

**SUPPLEMENTAL MATERIAL**

**Supplemental Figure 1**: Bivariate trait relationships between microanatomical, physiological, and stoichiometric traits delineated by year (red, grazed; blue ungrazed). Mean_X_BS (*BS_A_*), mean_X_Meso (*MS_A_*), mean_X_Vein (*V_A_*), mean_Xylem_Area (*X_A_*), mean_Photo (*A_n_*), mean_N (*N*), mean_C_N (*C*:*N*), MAP (mean annual precipitation), mean_temp (mean annual temperature), sum_PPT_jan_may (early season precipitation), sum_PPT_GS (growing season precipitation; these data include June – last sampling in August for both 2018 and 2019), mean_temp_GS (growing season temperature; these data include June – last sampling in August for both 2018 and 2019).

**Supplemental Figure 2**: Bivariate trait relationships between gas exchange and climate parameter data delineated by year (red, grazed; blue, ungrazed). cv_X_BS (*BS_A_*), cv _X_Meso (*MS_A_*), cv mean_X_Vein (*V_A_*), cv _Xylem_Area (*X_A_*), cv _Photo (*A_n_*), cv _N (*N*), cv _C_N (*C*:*N*), MAP (mean annual precipitation), mean_temp (mean annual temperature), sum_PPT_jan_may (early season precipitation), sum_PPT_GS (growing season precipitation; these data include June – last sampling in August for both 2018 and 2019), mean_temp_GS (growing season temperature; these data include June – last sampling in August for both 2018 and 2019).

Supplemental Table 1: Mean values and standard deviations for physiological, stoichiometric traits of *A. gerardii* and plot biomass for each location, year, and treatment.

**Supplemental Table 2:** Mean values and standard deviations for microanatomical traits of *A. gerardii* and for each location, year, and treatment

**
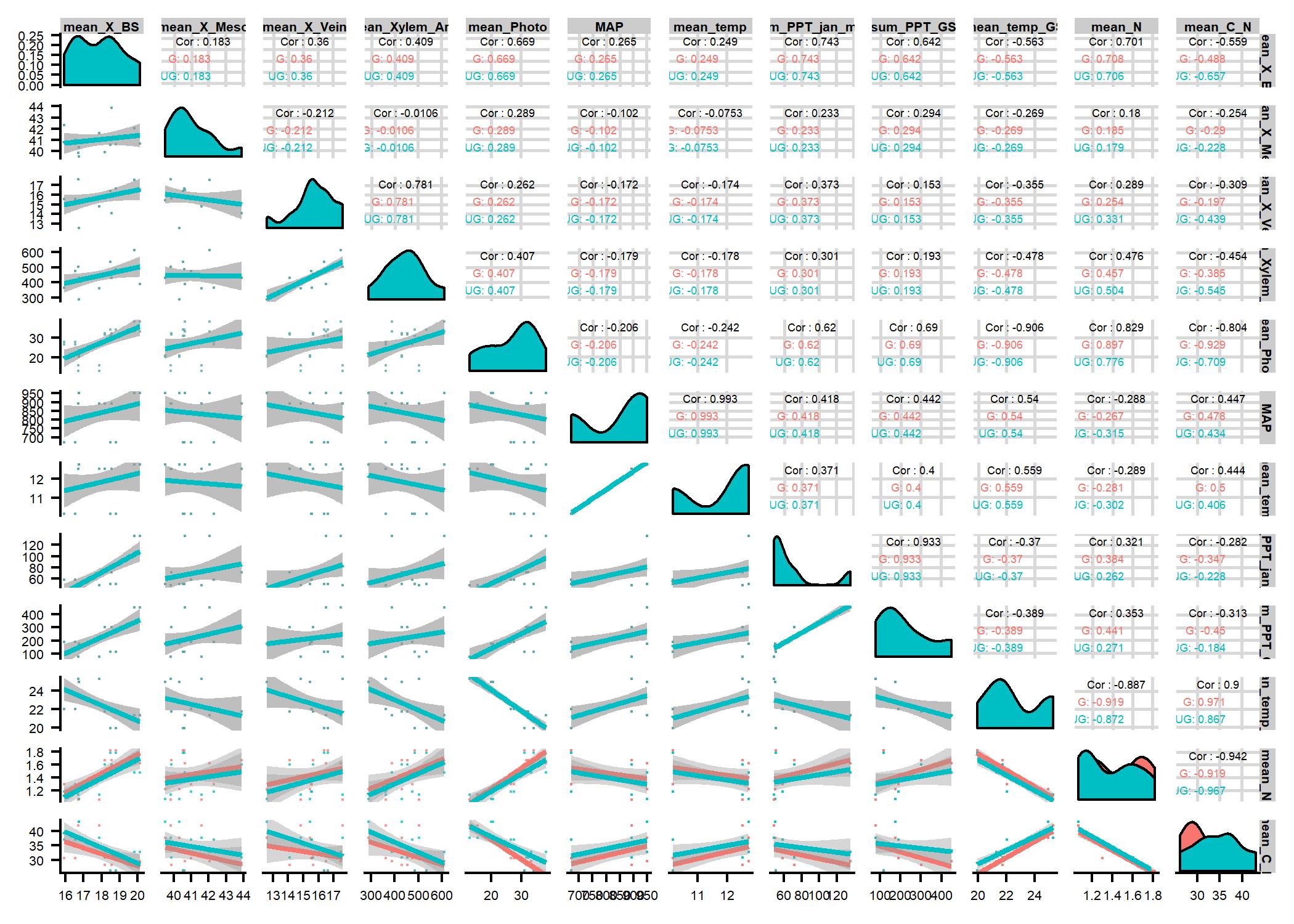
**

**Fig. S1**

**
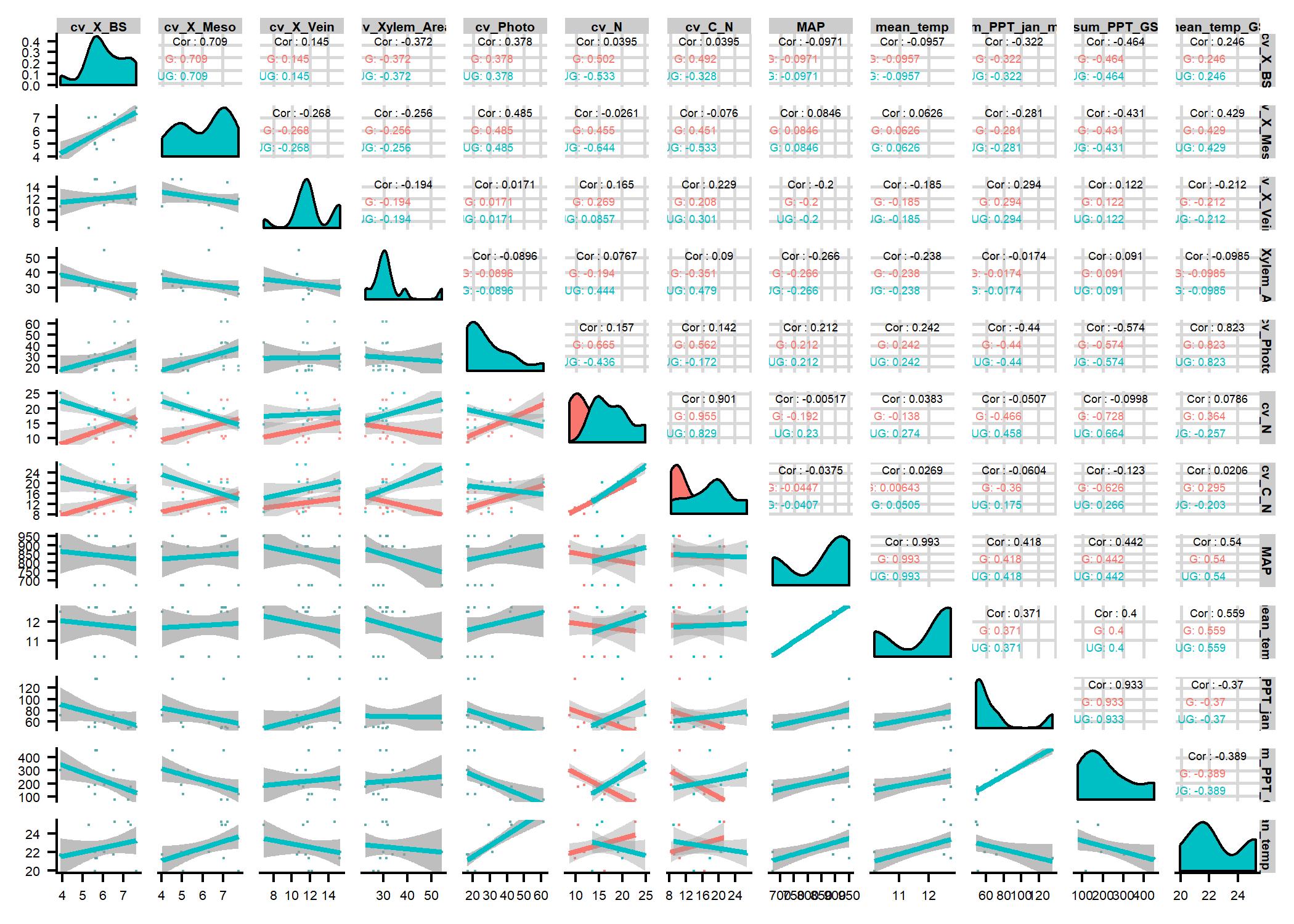
**

**Fig. S2**

**Table S1**

| Trait | Location | Grazed | | Ungrazed | |
| --- | --- | --- | --- | --- | --- |
|  |  | **2018** | **2019** | **2018** | **2019** |
| *A_n_* | FHPP | 20.77 ± 8.84 | 38.08 ± 8.11 | 20.15 ± 5.93 | 33.26 ± 5.87 |
|  | KPBS | 13.06 ± 8.01 | 31.70 ± 5.39 | 15.65 ± 6.59 | 32.31 ± 5.62 |
|  | PRP | 26.64 ± 8.45 | 34.08 ± 7.33 | 27.69 ± 6.98 | 30.98 ± 5.28 |
| *g_s_* | FHPP | 0.13 ± 0.07 | 0.32 ± 0.10 | 0.13 ± 0.07 | 0.36 ± 0.09 |
|  | KPBS | 0.10 ± 0.06 | 0.27 ± 0.06 | 0.12 ± 0.05 | 0.28 ± 0.07 |
|  | PRP | 0.19 ± 0.08 | 0.23 ± 0.05 | 0.18 ± 0.06 | 0.21 ± 0.04 |
| *E* | FHPP | 5.15 ± 1.98 | 7.88 ±1.75 | 4.09 ± 1.98 | 7.90 ± 1.63 |
|  | KPBS | 5.21 ± 3.09 | 4.97 ± 1.05 | 5.53 ± 2.94 | 5.55 ± 1.21 |
|  | PRP | 7.83 ± 2.95 | 4.14 ± 1.67 | 6.47 ± 2.37 | 5.27 ± 1.66 |
| *N*  *(%)* | FHPP | 1.22 ± 0.13 | 1.62 ± 0.16 | 1.07 ± 0.16 | 1.48 ± 0.30 |
|  | KPBS | 1.07 ± 0.24 | 1.67 ± 0.14 | 1.14 ± 0.18 | 1.60 ± 0.40 |
|  | PRP | 1.30 ± 0.15 | 1.81 ± 0.30 | 1.19 ± 0.23 | 1.78 ± 0.24 |
| *C:N* | FHPP | 37.63 ± 3.91 | 29.61 ± 3.11 | 42.99 ± 3.88 | 32.81 ± 5.85 |
|  | KPBS | 41.84 ± 8.92 | 28.29 ± 2.59 | 37.50 ± 7.61 | 30.89 ± 8.32 |
|  | PRP | 30.51 ± 2.55 | 26.26 ± 4.35 | 37.00 ± 7.61 | 26.47 ± 3.69 |
| *Biomass*  *(g m^-2^)* | FHPP | 174.04 ± 31.47 | 218.50 ± 44.18 | 492.66 ± 50.59 | 452.92 ± 182.80 |
|  | KPBS | 137.42 ± 43.39 | 255.34 ± 106.28 | 397.66 ± 135.71 | 599.92 ± 378.24 |
|  | PRP | 487.86 ± 110.49 | 781.44 ± 226.54 | 1096.86 ±317.67 | 878.94 ± 390.82 |

**Table S2**

| Trait | Location | Grazed | | Ungrazed | |
| --- | --- | --- | --- | --- | --- |
|  |  | **2018** | **2019** | **2018** | **2019** |
| *TMA*  *(µm^2^)* | FHPP | 148781 ± 32565 | 137626 ± 37691 | 135235 ± 37655 | 127206 ± 24573 |
|  | KPBS | 144733 ± 42711 | 180123 ± 51377 | 162726 ± 38373 | 159063 ± 49562 |
|  | PRP | 160728 ± 32409 | 187825 ± 97734 | 163832 ± 83120 | 152195 ± 36615 |
| *BS_A_*  *(%)* | FHPP | 16.733 ± 1.233 | 19.827 ± 1.127 | 16.728 ± 0.889 | 20.147 ± 1.134 |
|  | KPBS | 17.888 ± 1.180 | 18.554 ± 0.722 | 16.696 ± 1.209 | 18.195 ± 1.185 |
|  | PRP | 16.538 ± 0.927 | 18.494 ± 1.417 | 15.908 ± 0.964 | 18.816 ± 0.054 |
| *MS_A_*  *(%)* | FHPP | 40.326 ± 2.876 | 42.038 ± 1.912 | 39.52 ± 2.748 | 40.628 ± 2.2856 |
|  | KPBS | 41.589 ± 3.003 | 43.883 ± 1.763 | 39.827 ± 2.754 | 39.867 ± 2.108 |
|  | PRP | 41.004 ± 2.030 | 40.519 ± 3.143 | 42.29 ± 2.672 | 40.619 ± 2.045 |
| *BS:MS* | FHPP | 0.41767 ± 0.050 | 0.47226 ± 0.030 | 0.42484 ± 0.033 | 0.49897 ± 0.054 |
|  | KPBS | 0.43297 ± 0.050 | 0.42369 ± 0.028 | 0.42103 ± 0.041 | 0.45728 ± 0.034 |
|  | PRP | 0.40477 ± 0.037 | 0.46093 ± 0.067 | 0.3783 ± 0.041 | 0.46434 ± 0.036 |
| *V_A_*  *(%)* | FHPP | 12.599 ± 1.415 | 17.505 ± 2.668 | 15.438 ± 1.075 | 16.512 ± 1.958 |
|  | KPBS | 14.824 ± 2.174 | 14.105 ± 1.489 | 17.622 ± 2.070 | 15.649 ± 1.798 |
|  | PRP | 15.529 ± 2.370 | 16.383 ± 1.945 | 15.561 ± 1.681 | 16.644 ± 2.030 |
| *B_A_*  *(%)* | FHPP | 30.342 ± 3.453 | 20.631 ± 3.530 | 28.315 ± 3.339 | 22.712 ± 3.488 |
|  | KPBS | 25.700 ± 3.256 | 23.458 ± 1.822 | 25.855 ± 3.841 | 26.289 ± 3.192 |
|  | PRP | 26.928 ± 2.308 | 24.604 ± 2.632 | 26.241 ± 2.270 | 23.922 ± 2.594 |
| *X_A_*  *(µm^2^)* | FHPP | 289.40 ± 65.291 | 609.88 ± 190.12 | 430.21 ± 136.891 | 390.17 ± 111.797 |
|  | KPBS | 362.10 ± 113.592 | 431.25 ± 167.453 | 503.36 ± 149.089 | 453.59 ± 153.114 |
|  | PRP | 474.56 ± 141.986 | 534.18 ± 138.488 | 362.97 ± 197.666 | 486.46 ± 136.203 |
| *t/b* | FHPP | 0.047136 ± 0.008 | 0.03254 ± 0.004 | 0.042413 ± 0.008 | 0.038567 ± 0.005 |
|  | KPBS | 0.04455 ± .0010 | 0.04258 ± 0.008 | 0.041157 ± 0.007 | 0.039907 ± 0.007 |
|  | PRP | 0.040307 ± 0.007 | 0.03656 ± 0.005 | 0.050986 ± 0.011 | 0.040236 ± 0.006 |
